# Social, mobility and contact networks in shaping health behaviours and infectious disease dynamics: a scoping review

**DOI:** 10.1186/s40249-025-01378-6

**Published:** 2025-12-03

**Authors:** Zhifeng Cheng, Nick W. Ruktanonchai, Amy Wesolowski, Sen Pei, Jianghao Wang, Samantha Cockings, Andrew J. Tatem, Shengjie Lai

**Affiliations:** 1 School of Geography and Environmental Science, University of Southampton, Southampton, SO17 1BJ UK; 2 Department of Population Health Sciences, VA-MD College of Veterinary Medicine, Virginia Tech, Blacksburg, VA 24061 USA; 3 Department of Epidemiology, Bloomberg School of Public Health, Johns Hopkins University, Baltimore, MD 21205 USA; 4 Department of Environmental Health Sciences, Mailman School of Public Health, Columbia University, New York, NY 10032 USA; 5 State Key Laboratory of Resources and Environmental Information System, Institute of Geographic Sciences and Natural Resources Research, Chinese Academy of Sciences, Beijing, China; 6 Institute of Life Sciences, University of Southampton, Southampton, SO17 1BJ UK

**Keywords:** Social networks, Mobility and contact networks, Behavioural contagion, Multiplex networks, Generative agent-based models, Infectious disease modelling

## Abstract

**Background:**

The interconnectedness of human society in this modern world can transform localised outbreaks into global pandemics, underscoring the pivotal roles of social, mobility and contact networks in shaping infectious disease dynamics. Although these networks share analogous contagion principles, they are often studied in isolation, hindering the incorporation of behavioural, informational, and epidemiological processes into disease models. This review synthesises current research on the interplay between social, mobility and contact networks in health behaviour contagion and infectious disease transmission.

**Methods:**

We searched Web-of-Science and PubMed from January 2000 to June 2025 for research on health behaviour contagion and information dissemination in social networks, pathogen spread through mobility and contact networks, and their joint impacts on epidemic dynamics. This was first done by a preliminary literature screening based on predefined criteria. With potentially relevant publications retained, we performed keyword co-occurrence network analysis to identify the most common themes in studies. The results guide us to narrow down the reviewing scope to the social, mobility and contact network impacts on informational, behavioural, and epidemiological dynamics. We then further identified and reviewed the literature on these multidimensional network influences.

**Results:**

Our review finds that each network type plays a distinct yet interconnected role in shaping behaviours and disease dynamics. Social networks, comprising both online and offline interpersonal relationships, facilitate the dissemination of health information and influence behavioural responses to public health interventions. Concurrently, mobility and contact networks govern the spatiotemporal pathways of pathogen transmission, as demonstrated in recent pandemics. While traditional population-level models often overlook individual discrepancies and social network effects, significant efforts have been made through developing individual-level simulation-based models that integrate behavioural dynamics. With emerging new data sources and advanced computational techniques, two promising approaches—multiplex network analysis and generative agent-based modelling—offer frameworks for integrating the complex interdependencies among social, mobility and contact networks into epidemic dynamics estimation.

**Conclusions:**

This review highlights the theoretical and methodological advances in network-based infectious disease modelling and identifies critical knowledge and research gaps. Future research should prioritise integrating multi-source behavioural and spatial data, unifying modelling strategies, and developing scalable approaches for incorporating multilayer network data. The integrated approach will strengthen public health strategies, enabling equitable and effective interventions against emerging infections.

**Graphical Abstract:**

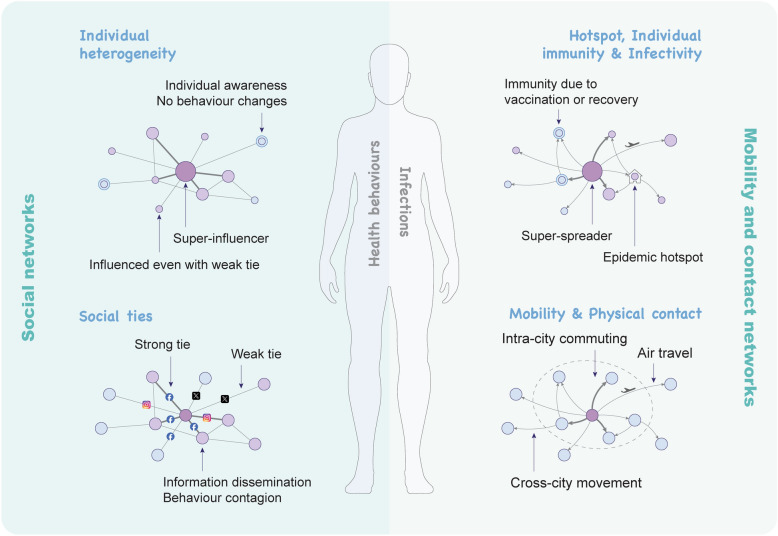

**Supplementary Information:**

The online version contains supplementary material available at 10.1186/s40249-025-01378-6.

## Background

Epidemics are increasingly evolving into pandemics in our interconnected and mobile world. Such global interconnectedness manifests through diverse individual interactions, from long-distance travel, direct contact, and face-to-face communication to virtual connections via emails, video calls, and social media. These interactions collectively form dynamic networks that evolve as interpersonal relationships develop and fade, not only facilitating information transfer and behaviour propagation but also driving pathogen transmission. Importantly, *human mobility and contact networks* (mobility and contact networks hereafter), a particular form of geographical network summarising human movement and physical contact, fundamentally shape patterns of infectious disease transmission [1]. As human hosts travel and interact physically, pathogens can be carried and transmitted, bridging geographical distances [2, 3] and triggering local outbreaks across different communities [4]. Consequently, public health authorities implement various containment measures, including contact tracing, quarantines, and even travel restrictions and social distancing to mitigate or contain outbreaks of infectious diseases [5–9].

The success of such measures, however, depends heavily on individual acceptance and compliance, which varies widely and is affected by broader systems of social interactions and personal relationships (referred to as *social networks*) [10–12]. Within social networks, ties between people, whether through in-person or online interactions, enable knowledge, awareness, and cognition to spread across individual, local, and global scales, transcending geographic boundaries [10, 13]. This information diffusion drives behaviour contagion between individuals both nearby and distant [14], fostering collective health behaviours [15–19] and the trajectory of infectious disease transmission [20–22]. For example, individuals may adopt preventive measures due to more intensive social connections to outbreak-affected areas, or conversely, develop vaccine hesitancy due to exposure to misinformation about vaccine efficacy and safety [20, 23].

The COVID-19 pandemic has underscored the critical need to integrate insights and approaches from epidemiology, social sciences, geography, and network sciences to deepen our understanding of the interplay between social, mobility and contact networks [23–25]. While epidemiological models have proven invaluable in combating a pandemic [26–28], they often fall short in capturing the behavioural and social dynamics that fundamentally shape the transmission patterns of infectious diseases, particularly for human-to-human infections [29]. These dynamics manifest in various ways—from the resumption of outdoor activities and vaccination acceptance to compliance with public health policies—all of which are influenced by social connections, information flow, and travel frequency that vary across space and time [20, 23, 30].

Fortunately, increasing availability and variability of diverse human activity footprint data enable a comprehensive understanding of complex social interactions for infectious disease dynamics. On the one hand, traditional disease models solely based on mobility and contact networks already have a rich selection of data sources. Those include records from cellular signalling [31–33], GPS positioning [34], and public transportation [2, 3], enabling investigation of disease transmission risks attributed to multi-scale human mobility from intra-city, inter-city, to international [1]. Further, new data sources provide opportunities to measure multi-dimensional information simultaneously, especially those related to societal and social interactions in addition to mobility and contact. For instance, mobile phone data [35] and geotagged social media data [36] can be used to infer both human mobility patterns and friendship network structure. Mobile health code records, like data from the NHS COVID-19 app, can reflect both close physical contact and social relationships [37].

Despite sharing similar theoretical foundations of complex networks and the potential of new data sources for integrated analysis, current research still lacks a comprehensive synthesis of how information dissemination, behavioural contagion, and pathogen transmission interdependently influence disease dynamics. This knowledge gap hinders the precise formulation and effective implementation of public health interventions for emerging infectious diseases. This review aims to bridge these gaps by synthesising current research on the roles of social, mobility and contact networks in shaping health behaviours and mediating infectious disease transmission. We first define and contextualise these networks, introducing their formation, evolution, and structural characteristics. Then, we elucidate how social networks drive information dissemination and behaviour contagion, extending these insights to pathogen transmission dynamics. We further review existing infectious disease models, ranging from population-level compartmental and metapopulation models, to individual-level agent-based models and multiplex networks, and finally emerging generative agent-based models. We highlight their strengths and limitations for considering individual behavioural discrepancies and their ability to integrate social, mobility and contact networks. Finally, we identify critical knowledge gaps and propose future research directions to foster more integrated and effective public health strategies, emphasising the crucial synergy between behaviours, information, and diseases.

## Methods

### Search strategy

**Fig. 1:**
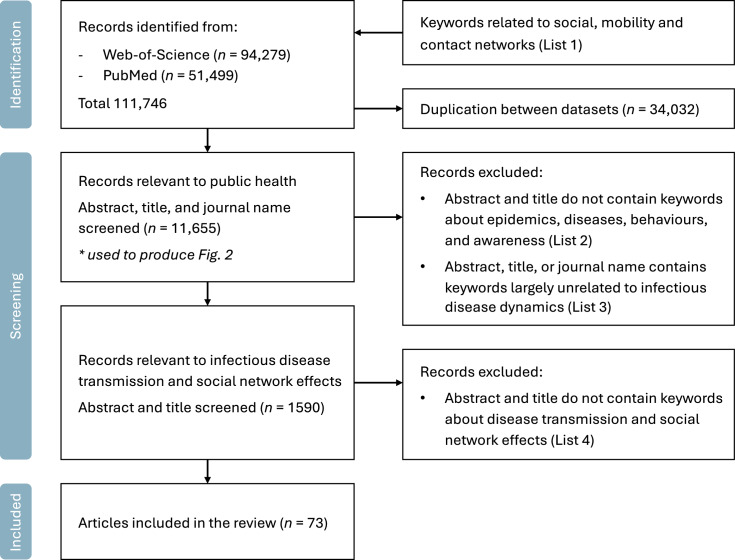
Flowchart of literature searching and selection

The literature review involved a systematic search of bibliometric databases Web-of-Science and PubMed, retrieving records from January 2000 to June 2025. As shown in Fig. 1, we gathered papers containing terms relative to social, mobility and contact network in their titles or abstracts (List 1): social network, social interaction, human mobility, mobility network, human contact, physical contact, and contact network. After deduplicating records across databases, a total of 111,746 publications have been found.

### Inclusion and exclusion criteria

We selected papers potentially relevant to public health applications by searching their abstracts for any of the keywords in List 2. The list consisted of three categories of public health, respiratory infections, and human behaviours, including: epidemi*, pandemic*, disease*, infect*, COVID, coronavirus*, nCov, influenza, malaria, HIV, contact tracing, behavio*, interven*, contagio*, perception, awareness, belief, misinformation, rumor, and citizen science. To restrict publications more precisely to our reviewing scope, we further excluded records by filtering out papers if their titles, abstracts, or journal names contain keywords largely irrelevant to our focus, such as ageing, chronic, or ecological (List 3, full list presented in Supplementary Materials).

### Evidence extraction and analysis

**Fig. 2:**
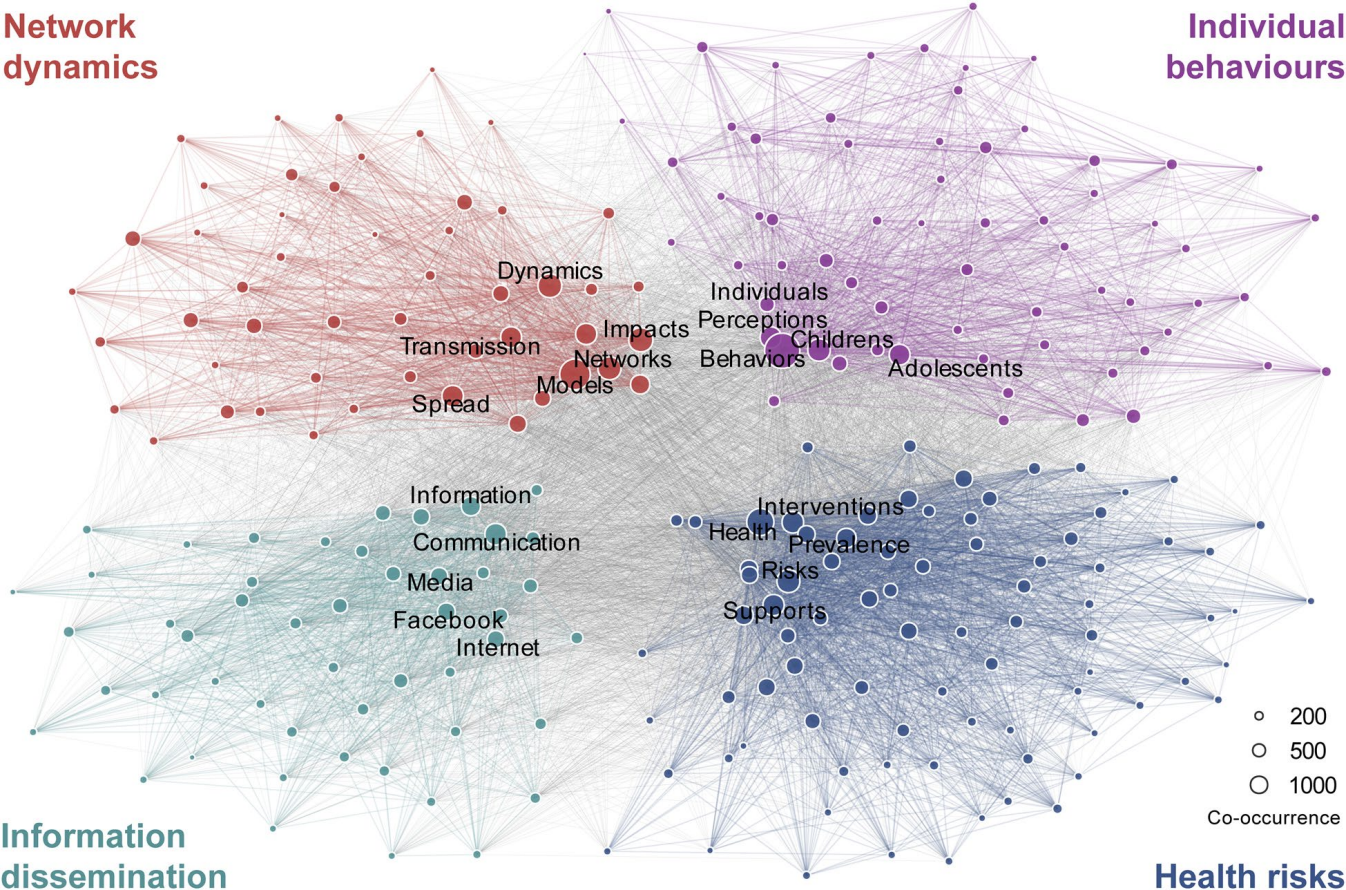
Keyword co-occurrence network of research on public health applications of social, mobility and contact networks. The network is constructed using the “Keywords.Plus” field in the metadata of publications indexed in Web-of-Science up to June 2025, comprising papers that probably apply social, mobility and contact networks to public health practice. Community detection is performed using the Louvain algorithm. The network visualisation displays the top 250 keywords that occur at least 20 times in the dataset

To gain initial insights from existing studies, we investigated the keyword co-occurrence network using bibliometric metadata from Web-of-Science publications. The network is constructed using the “Keywords.Plus” field, where any pair of keywords co-occurring in a paper constitutes a link in the network. We performed the Louvain community detection algorithm and identified several themes that are highly concentrated in existing studies (Fig. 2 and subsection “Keyword co-occurrence network and major research themes”). Based on these findings, we determined the reviewing scope as the social, mobility and contact network impacts on informational, behavioural, and epidemiological dynamics. We kept papers potentially relevant to infectious disease spreading or social network influences by searching their titles and abstracts with keywords (List 4): spread*, transmission, transmit*, peer influence, peer effect, network effect, and network intervention. This step shrinks the candidate literature size to 1590 records, of which 73 were finally included in this review.

## Results

### Keyword co-occurrence network and major research themes

The community detection of keyword co-occurrence network reveals four primary themes, one of which brought together keywords related to basic network dynamics like models, impacts, and transmission, and the other three could be categorised as individual behaviours, health risks, and information dissemination (Fig. 2). Specifically, the individual behaviours category includes keywords including perceptions, behaviours, and children, emphasising cognitive processes and behaviour adoptions, with some particular focus on children and adolescents. Information dissemination includes keywords like communication, media, and Facebook, implying the importance of digital techniques and social media data sources in relevant studies. Finally, health risks are represented by health, interventions, prevalence, etc., showing disease transmission risks and public health interventions. The remainder of this review was organised based on these findings. We first review behavioural contagion and (mis)information dissemination and its resulting heterogeneity in individual awareness and diverse disease infection risks, and then extend their influence to the infectious disease dynamics.

### Health behaviour contagion and information dissemination

#### Behavioural contagion via social networks

Social networks profoundly shape behaviour contagions through both strong or weak interpersonal ties connected, as people acquire what others think and behave via face-to-face or virtual communication. This social learning process generates perceived consensus and collective behaviours, which in turn trigger individual behavioural changes [10, 14]. The potency of such network effects is particularly evident in health-related behaviours. Studies found that having a friend who quit smoking reduces one’s odds of smoking by 57% [15], while having an obese friend increases one’s probability of obesity by 36%, escalating to 67% if the person is a spouse [16]. Similar contagion dynamics have been observed in alcohol consumption [38, 39] and drug intake [40]. Quantitative evidence from Mir Ali’s research demonstrates that a 10% increase in peer engagement amplifies an adolescent’s likelihood of drinking by 3% [41], smoking by 4% [42], and marijuana use by 5% [43].

However, the increasing prevalence of certain behaviours cannot be entirely attributed to the influence of social networks alone. Homophily, the tendency of individuals to imitate peers similar to themselves, also plays a critical role in behaviour patterns [44–46]. Unlike influence-driven contagion, which tends to be directional, self-reinforced, and rapid, homophily reflects the network structure where nodes mutually affect each other. Distinguishing homophily-driven behavioural diffusion from influence-driven contagion is vital for understanding the mechanisms of behavioural changes. For instance, Aral et al. show that homophily accounts for over 50% of the observed product adoption decisions [47]. In addition, behavioural contagion is more likely to be a complex process, where multiple-source exposures are required for adoption [48]. In this context, highly clustered strong ties among mutual friends within homogenised clusters are particularly effective as repeated interactions reinforce perceptions and affirm group identity. On the downside, however, homophily may impede cross-group interactions [49], limiting information dissemination to peripheral minorities [50] and aggravating disparities in perceptions across different social network groups [51].

The influence of homophily becomes even more complex when considering social network formation, where peer effect and peer selection occur symbiotically. These dual processes manifest as individuals both choose whom to befriend and whose behaviour to imitate [52]. The peer effect drives behavioural changes to align with peers, while peer selection leads to forming ties with those exhibiting similar behaviours. This dynamic can be understood through the concepts of “induced homophily” (arising from structural proximity, such as shared schools or workplaces) and “choice homophily” (stemming from selective preferences based on characteristics like age and gender) [45]. Both mechanisms contribute to social network tie formation and can confound estimates of behavioural influence if not properly accounted for [53–55].

The principles of behavioural contagion have been effectively harnessed for targeted health interventions [18, 56]. Studies have demonstrated that propagating norms through social media friends is more effective than direct information delivery in encouraging physical activity and fostering positive attitudes and self-efficacy [19]. Similar benefits have emerged in anti-obesity campaigns [57]. While social media platforms are effective mediums for delivering health behaviour interventions [58], the outcomes vary substantially across health topics and participant characteristics, necessitating tailored strategies for maximum impact [59].

#### Knowledge and misinformation dissemination in online social networks

In this digital age, behaviour and information dissemination transcend geographic boundaries of neighbourhoods, schools, and communities to reach global scales. Online social networks amplify truth and scientific knowledge, but also misinformation, with topics like climate change beliefs [60], bombshells [61], and infectious diseases [62] frequently accompanied by rumours, fake news, or scepticism propagating through online social media. Analogous to the mechanism of behaviour contagion, online (mis-)information diffusion is often driven by selective exposure, which promotes the formation of homogeneous network clusters [63]. These clusters exacerbate misinformation circulation within segregated groups, creating echo chambers that reinforce false narratives [64]. Automated agents, or bots, further complicate the landscape by deliberately setting about misinformation, amplifying threats to public health and safety [65].

Infectious diseases and vaccination represent two of the most widely discussed themes in health misinformation [66]. Early evidence from Ebola outbreaks suggests that misinformation is associated with reduced trust in formal medical care, diminished perception of disease risks, and decreased self-protective actions [67]. The COVID-19 pandemic has reaffirmed these findings, with scholars documenting a surge in fake news and rumours concurrent with pathogens spread, leading to the coinage of “infodemic” to describe the proliferation of misinformation [24, 25]. Such prevalence of misinformation tends to perpetuate and intensify incorrect beliefs, particularly among populations with lower education attainment and stronger anti-science political orientations.

The other critical consequence of misinformation is vaccine hesitancy, defined as the delay or refusal of vaccination despite its availability [68, 69], which has been documented across a variety of infectious diseases [70, 71]. This hesitancy stems from concerns about vaccine efficacy and safety, mistrust in governments and healthcare professionals, and varying perceptions of disease risks [72, 73]. Social networks play a pivotal role in this phenomenon, as individuals’ willingness to get vaccinated fluctuates with the attitude of their family members and friends, reflecting the impact of homophily [11, 74]. Information flows significantly influence vaccination behaviour: negative information about vaccine side effects significantly reduces vaccine acceptance and prolongs outbreaks, while information on infection prevalence boosts vaccination rates [75]. Consequently, the proliferation of anti-intellectualism [12, 23] and vaccine misinformation [76–78] through social media can undermine vaccination campaigns, eventually contributing to increased morbidity and mortality during health crises.

### Impacts of social, mobility and contact networks on epidemics

**Fig. 3:**
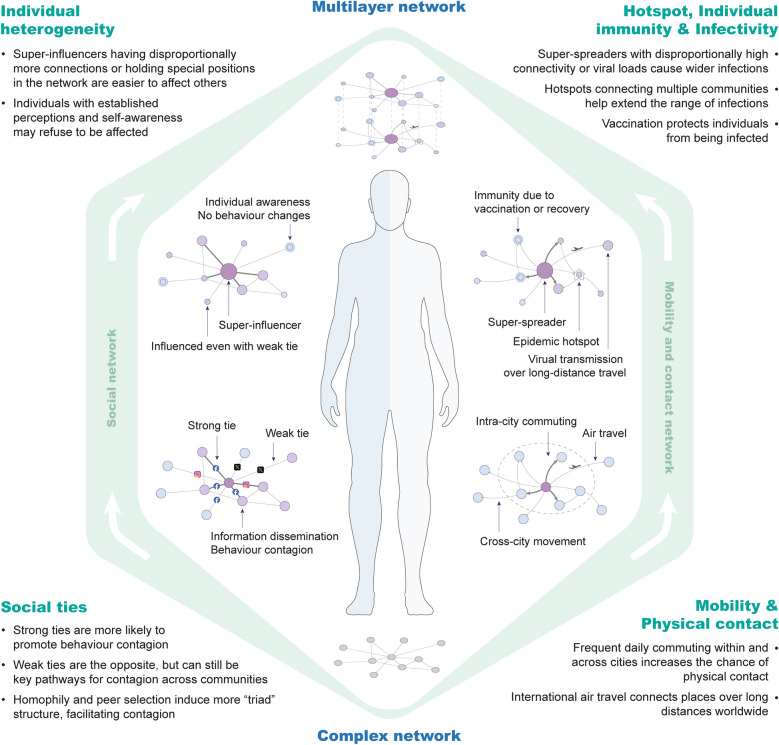
The potential impact of social, mobility and contact networks on infectious disease transmission

While social networks primarily facilitate the spread of health behaviours and information, mobility and contact networks may directly enable disease transmission through physical proximity, together with individual heterogeneity in immunity and behaviours as well as biological and environmental factors (Fig. 3). This section examines the distinct roles of these networks in shaping infectious disease patterns and reviews existing efforts to integrate them into disease modelling frameworks (Table 1), ultimately advancing our understanding of how human interactions across multiple dimensions influence epidemic dynamics.

**Table 1:** Strengths and limitations of different networks and models for modelling health behaviour contagion and infectious disease transmission

Network types and models		Strengths	Limitations	References
Social networks	Regression models	• Direct relationship between individuals’ and friends’ behaviours • Longitudinal cohort study can reveal causality	o Surveys are costly, limited to small samples, and potentially biased to the general population o Cross-sectional design makes strong causal inference challenging when rounds of surveys are limited	[10, 15–17, 20, 38–43, 79, 90]
	Network models	• Explicit dynamics of information and behaviour contagion process within social networks • Feasible for designing targeted network intervention	o Real-world validation needs rigorous pre-experimental design, e.g., randomised controlled trial (RCT) o Simulations and synthetic samples are widely used when real data are unavailable or static	[14, 19, 23, 57, 63–65, 73–75, 77, 80, 84]
Mobility and contact networks	Compartmental/metapopulation models	• Simple framework allows rapid assessment of disease transmission • Low data requirement with no need for human activity records, or rely on human mobility across regions only	o Homogeneous mixing assumption deviates from reality o Individual behavioural discrepancies are mostly ignored	[1, 8, 9, 26, 28, 32, 93, 95, 101, 103, 118, 119]
	Agent-based models (ABMs)	• Individual-level modelling captures heterogeneity in human behaviours • Reflect real-world complexities	o High in data requirements, computing resources, and output uncertainty	[126, 127, 150]
Integrated social, mobility and contact networks	Multiplex network-based models	• Explicitly connect spreading processes of information, behaviours, and diseases • Flexible, expandable, multi-layer structures can better resemble real-world complexities	o Detailed individual-level data on both social and physical interactions is hard to obtain o Primarily simulation-based, with limited real-world application	[134–149, 154–157]
	Generative agent-based models (GABMs)	• Generative agent acts resembling human thoughts and behaviours in the real world • Accept information directly instead of predefined rules • Allow dynamic adjustment	o Challenging in model explainability and reproducibility	[151–153]

#### Social interaction and pathogen spread

Understanding information dissemination and behavioural contagion provides crucial insights into preventing and controlling pathogens, from those transmitted through direct physical contact (e.g., HIV and syphilis) to those spread via vectors or droplets (e.g., malaria and influenza). In sexually transmitted infections (STIs), social networks significantly influence transmission risk, as members of sexual networks or drug user communities often share similar norms, risk behaviours, and mutual social support [21]. For instance, people who inject drugs (PWID) commonly endorse sharing paraphernalia such as cookers or needles, which facilitates pathogen spread, while individuals who share cookers are less likely to accept injection [79]. In terms of protective behaviours, more discussion about pre-exposure prophylaxis (PrEP) with friends would significantly increase network members’ PrEP knowledge, attitudes, norm perceptions, and self-efficacy, uplifting the percentage of PrEP adoption from 3 to 11% [80]. Based on the theories of weak ties and super-influencers [81–83], as demonstrated in Fig. 3, social network interventions targeting “bridging” individuals who connect multiple communities have been recommended to effectively reduce risk behaviours like needle-sharing and unprotected sex [84], which helps access populations unreached by typical clinical and public health efforts [85].

Social networks can similarly shape the dynamics of vector-borne diseases (VBDs) such as malaria. Exposure to disease-related information through social networks significantly alters individuals’ willingness to adopt preventive measures. For instance, broadcasting malaria information would increase the use of insecticide-treated bed nets and antimalarial drugs during pregnancy, where those who did not receive information were 36% and 23% less likely to adopt, respectively, as reported by a study conducted in sub-Saharan Africa [22]. In regions with limited public health surveillance infrastructure, participatory approaches, including citizen science programs, have emerged to engage the public in VBDs monitoring and control [86–88]. These approaches foster broader social interactions, expanding networks and promoting health knowledge and behaviours among populations initially hesitant or resistant to interventions such as indoor residual spraying for malaria control [89].

Social networks play a pivotal role in shaping responses to not only endemic or epidemic diseases but also global pandemics, as demonstrated during SARS-CoV-2 transmission in the early 2020s. Research indicates that individuals’ attitudes towards public health measures can be remarkably influenced by their peers’ opinions and behaviours. For example, lockdown or reopening policies implemented in one area can create ripple effects in neighbouring regions, influencing residents’ decisions to restrict or increase outdoor activities [90]. While policies are designed with expected behavioural changes in mind, coordinating public compliance remains challenging across different countries and cultural contexts [91]. Public non-compliance with governmental recommendations often stems from underestimating disease severity, doubting measure effectiveness, and particularly from the experiences and attitudes of their relatives and friends. Studies found that individuals having more social media connections with early COVID-affected regions (e.g. China and Italy) demonstrated greater willingness to reduce outdoor activities, especially in areas with a higher education level and a lower fraction of climate change deniers [20]. Meanwhile, Intense social bonding with family and with more groups correlates with better mental well-being and higher willingness to adopt health behaviours like distancing or wearing masks [92]. However, in later pandemic stages, exposure to anti-vaccination views or adverse events remarkably increased vaccine hesitancy [23], illustrating how network influences evolve throughout a pandemic.

#### Human mobility and contact affect respiratory pathogen spread

Human mobility and contact networks play an exclusive role in respiratory disease transmission. Unlike pathogens transmitted via blood, vectors, or contaminated water and food, the spread of respiratory infectious diseases critically depends on droplets, aerosols, or close contact between individuals [1, 93]. Consistent with the theory of strong and weak ties in social networks [81], the type and intensity of human close contacts shape respiratory pathogen dynamics. For instance, within schools, student interactions in the same class induce higher influenza transmission probabilities compared to interactions between different classes or grades, with school-aged children often acting as intermediaries transmitting infections from schools to households [94].

At the urban scale, daily commuting and modern transportation facilitate pathogen spread between communities. Research shows that commuting volume strongly correlates with the prevalence of influenza-like illness [95], with crowded public transportation systems further amplifying transmission risks [96]. Beyond volume, pathogen transmission also correlates with the type of travel destinations. Visits to venues that bridge multiple communities, such as retail and recreation centres, workplaces, and transit hubs, promote more severe epidemics [97]. Since human movement usually faces constraints of spatial costs, exhibiting scale-free decay of visitation with increasing distance [98, 99], it is commonly observed that infections first spread within densely connected urban centres before extending to more distant regions [100].

Air travel serves as a key bridging tie in mobility networks, facilitating rapid spread of respiratory pathogens across countries [2, 3]. For instance, during the 2009 influenza A H1N1 pandemic, even stringent travel restrictions that reduced air traffic from the disease hotspot by 40% only delayed the international spread by three days [101]. The recent COVID-19 pandemic further emphasised air travel’s pronounced role, as it quickly reshaped SARS-CoV-2 transmission patterns by shifting the main contributors from the first-reporting country to the most interconnected ones, particularly for highly transmissible variants like Delta and Omicron [102]. This phenomenon underscores the interplay between virus infectivity and global connectivity. Consequently, non-pharmaceutical interventions such as travel restrictions have been widely implemented to reduce the density of mobility and contact networks [6, 26, 103, 104]. These measures contain global circulation of not only SARS-CoV-2 but also H1N1, H3N2, and B/Victoria influenza viruses [105]. Furthermore, travel restrictions become more effective when coupled with close contact tracing to identify and interrupt specific transmission pathways [106, 107]. This understanding has been validated across multiple outbreaks, including SARS [4, 5, 108, 109], influenza, and COVID-19 [110, 111].

Targeted interventions focusing on super-spreaders [112, 113], individuals with disproportionately high infectivity, can significantly curtail outbreaks. Unlike super-influencers who occupy strategic positions in social networks [82, 83], super-spreaders may be either structurally prominent with numerous contacts or those who have higher viral loads and generate above-average numbers of secondary cases [114]. During the 2003 SARS outbreak, a small number of super-spreaders induced the majority of early cases in Singapore and Hong Kong, China [5, 108], while COVID-19 documented more than twice the number of super-spreading events in the literature [115]. These findings highlight the importance of considering individual heterogeneity in transmission dynamics across mobility and contact networks when measuring epidemiological characteristics, predicting risks, and developing response strategies.

### Epidemiological models integrating social, mobility and contact networks

#### Population-level modelling with mobility and contact networks

Modelling strategies largely determine the extent to which and how well we can translate knowledge of social, mobility and contact networks into more accurate estimation and prediction of infectious disease dynamics. Traditional population-level models, particularly compartmental models, have long served as foundational tools in epidemiology. These models—such as the Susceptible–Exposed–Infected–Recovered framework—represent individuals as belonging to discrete epidemiological states, transitioning between them based on pre-defined rates or probabilities [26]. However, a key limitation of this approach is its assumption of homogeneous mixing, whereby all individuals are equally likely to interact with one another regardless of social or spatial constraints in the real world [116, 117]. Although its relatively simple framework remains valuable in early rapid assessments of disease transmission, the mechanistic rules of human actions and virus spreading are insufficiently considered, especially in terms of the regularity in human mobility and contact patterns and the heterogeneity in individual preventive behavioural choices [98].

As a preliminary effort to avoid such deficiencies, meta-population models explicitly extend compartmental frameworks into geographical space by segmenting the population into interconnected subpopulations (e.g. communities, cities, or regions) [118, 119]. These subpopulations are linked by migration rates or mobility flows, allowing for dynamically redistributing susceptible and infected groups across locations and updating the probability of risk contact at each time step. By explicitly modelling spatial heterogeneity, meta-population models more accurately capture the spatiotemporal dynamics of disease transmission and the effects of interventions such as travel restrictions or regional lockdowns.

#### Incorporating behavioural feedback and social influences

Beyond mobility, social and behavioural factors are also worth being incorporated into disease modelling [120]. A key insight from economic epidemiology is the concept of prevalence-elastic behaviour—the tendency of individuals to adopt protective behaviours (e.g., mask-wearing, social distancing) more readily as disease prevalence increases, and to relax those behaviours when risk perception declines [121–123]. For example, people are more willing to reduce risk behaviours like needle sharing when HIV infection increases. Comparatively, when the infections are low, individuals are less motivated to adopt protective behaviours. This trade-off between infection risks and personal health/economic benefits has also been discussed in other interventions, including vaccination and social distancing [124].

Such a behavioural feedback loop underscores the necessity of modelling the co-evolution of disease transmission, information diffusion, and behaviour adoption. However, previous reviews have pointed out that existing models tend to rely heavily on rational-actor assumptions derived from behavioural economics, instead of insights from psychology or sociology that emphasise social learning, imitation, and peer influence [125]. Although cognitive contributions to behaviour adoption, like the aforementioned trade-off between perceived risk and costs of preventive behaviours, are well acknowledged, the social network structures through which individuals acquire, interpret, and act upon health information are rarely modelled explicitly, especially for infectious diseases [29]. In other words, very few existing papers made an explicit explanation of the “social network impact” on human behaviours for disease dynamics, relying instead upon “social impact” only. This disconnection stems partly from disparities in data availability, scales, and timeliness. Macro-level or population-level mobility data often lack individual details, while micro-level or individual-level data on social and informational ties are difficult to obtain due to privacy concerns in the real world. As a result, the interplay between social, mobility and contact networks remains underrepresented in traditional population-level models.

#### Individual-level modelling: coupling social, mobility and contact networks

Theoretical advances and emerging computational techniques demonstrate the potential of integrating intrinsically linked social factors into infectious disease models, where one key amendment is to shift the modelling strategy from “top-down” (population-level) to “bottom-up” (individual-level). In the “top-down” approach such as compartmental or meta-population models, the disease dynamics are estimated by considering interventions that are imposed at the population level, primarily affecting immunity and the probability of physical contacts. In contrast, “bottom-up” models simulate emergent dynamics from individual decisions and interactions, accounting for the effects of social, mobility and contact networks simultaneously. Table 1 summarises the strengths and limitations of different network types and modelling approaches for infectious diseases.

Agent-based models (ABMs) usually serve as a basic choice for “bottom-up” modelling [126]. Rather than simulating macro-level transitions among aggregated population states (e.g., shifts between susceptible and infected populations), ABMs model individual agents with heterogeneous attributes, perceptions, and behavioural rules. Agents can exhibit diverse behaviours like mobility choices, adoption of self-protective actions, and vaccine uptake [127, 128]. It also simulates interactions among individuals and allows their behaviours to evolve over time, whereby the collective behaviours of a set of representative agents reflect heterogeneous population composition. Traditionally, ABMs operate using predefined behavioural rules, but such approaches struggle to capture the stochastic nature and dynamic changes of real-world behaviours. Consequently, ABMs need to be extended to account for these complex, multilayered interactions.

#### Multilayer and multiplex networks in ABMs

One key extension of AMBs involves embedding agents within multilayer or multiplex networks to more realistically simulate interactions across physical, social, and informational domains [129]. Multilayer networks can represent different types of interactions, such as local and long-range mobility, co-infection dynamics, or time-varying contact patterns using separate but interconnected layers [130–133]. However, when the focus is on how information dissemination influences individual behaviours that in turn affect disease transmission, multiplex networks are especially useful [134, 135].

Multiplex networks differ from multilayer networks mainly in that they require a one-to-one correspondence of nodes across different layers, so that the effect of network contagion in one layer can cascade to other layers at the individual level. A common application, for instance, is the two-layer network model representing awareness diffusion and disease transmission. Such models usually hypothesise that susceptible individuals who become aware of infection risks through social networks may adopt protective behaviours (e.g., isolation, hygiene), thereby lowering their likelihood of infection. Infected individuals may actively alter their own mobility or contact patterns, in turn, indirectly influencing the awareness of their social network peers. These dynamics create feedback loops between awareness and epidemic spread, potentially reducing outbreak sizes when awareness diffusion and the prevalence of self-protective behaviours reach a critical threshold [136–139]. Furthermore, research has revealed the distinct effects of global and local awareness on disease dynamics. While local awareness typically propagates through peer networks within communities [140], global awareness disseminated via mass media or major social media platforms often proves more effective at suppressing disease spread by providing broader, society-wide perspectives [141–143].

Despite their utility, two-layer multiplex networks remain insufficient to fully resemble the real-world complexities of human behaviour. Individuals may exhibit diverse responses to the same information, influenced by factors such as personal beliefs, socio-demographic differences, and social context. To account for these variations, studies have proposed several model refinements by allowing variable vertex activities or expanding multiplex networks from two layers to three or more [144]. For instance, Rizzo et al. considered reduced activities from both infected people (due to illness or quarantine) and susceptible individuals (due to self-protection) [145]. Similarly to this concept, Song et al. introduced a weighted co-evolving multiplex network in which individuals can rewire connections to avoid physical contact with the infected [146]. Those behavioural changes can increase the epidemic threshold and decrease the fraction of infection.

To further incorporate enhanced awareness and competing information dynamics, Zhu et al. considered a more realistic situation that individuals are unwilling to share the information even if they have established perceptions about the disease [147]; while He et al. introduced the competition between rumour and knowledge diffusion on the information layer, reflecting the real-world competition between misinformation and verified health communication [148]. These extensions and refinements advance our ability to better simulate the interplay between information diffusion, behaviour adaptation, mobility/contact changes, and disease transmission. By capturing such coupled dynamics, multiplex network-based ABMs offer deeper insight than simpler frameworks into the nuanced dynamics of infectious disease spread in heterogeneous populations [149].

#### Stochastic agent-based models and generative agents

A further evolution of traditional rule-based ABMs involves the incorporation of stochastic processes, allowing models to account for the randomness and variability of human behaviours, which is far beyond the explanation of pre-defined rules or probabilities. Stochastic ABMs introduce randomness into individuals’ choices and interaction patterns while ensuring coherence at the population level. For example, Hoertel et al. built a stochastic ABM for modelling COVID-19 spreading in France [150]. They constructed a synthetic population where each agent has different demographic attributes (e.g., age, gender), household structure, and social contact patterns, such as activity sequence over the day (work, school, family, etc.) and co-location probability. These attributes were sampled and matched coordinating with national statistics, enabling realistic simulations of contact networks and transmission pathways.

The recent advent of large language models (LLMs) has provided further opportunities for creating generative agents—autonomous, language-driven individuals capable of making context-aware decisions based on evolving information [151]. Rather than determining certain assumptions about what behavioural dimensions to include and how each human would act, generative agent-based modelling (GABM) accepts diverse information directly and each agent makes their own decision-making on behavioural changes based on existing knowledge and the social environment, which can be defined using user prompts and system prompts, respectively [152, 153]. More importantly, researchers can pass new information (e.g., public health messages, news updates, or peer behaviours) to the agent at each time step, enabling dynamic responses that more closely resemble real-world behaviours over time. By combining stochastic behavioural diversity with natural language processing capabilities, GABM offers a powerful tool for exploring the cognitive and social dimensions of epidemics, especially in complex, dynamic environments where traditional rule-based approaches fall short.

#### From simulation to real-world application

Although ABMs have long been a proven approach for disease transmission modelling, their variants explicitly introducing individual awareness and social network impacts, including multiplex networks and GABM, remain primarily simulation-based with limited validations of real-world scenarios. This is largely due to the demanding data requirements to calibrate, validate, and apply such models, particularly those incorporating detailed behavioural, social, and mobility dynamics. Nonetheless, researchers have begun to apply these theoretical modelling frameworks by integrating diverse empirical data sources, including socioeconomic indicators, mobility traces, contact matrix, epidemiological surveillance, and digital behaviour signals [154].

For instance, Lima et al. leveraged call detail record (CDR) data to simultaneously extract the mobility matrices and communication networks among individuals [155]. Their multiplex network modelling demonstrated that disease prevention information through social networks, including hygiene practices and vaccination campaign notices, could effectively contain virus spread. Data-driven analysis from Scatà et al. further incorporated Google Trends data and socioeconomic conditions into a Zika virus transmission model. Their work revealed that individual heterogeneous awareness of diseases, coupled with attention decay, would remarkably alter Zika virus transmission dynamics, emphasising the importance of timely and targeted communication strategies [156]. Similarly, studies of influenza-like illnesses have shown that effective information dissemination can reduce outbreak magnitude by promoting early behavioural interventions [157]. All of these underscore the potential and necessity of linking theory-driven models with empirical data, moving beyond simulation and toward evidence-based forecasting, scenario testing, and intervention planning.

## Discussion

Social, mobility and contact networks mediate human behaviours and infectious disease dynamics. Although substantial progress has been made in understanding behaviour contagion and pathogen transmission independently, research integrating these processes remains limited. This gap is particularly critical as online social media increasingly shapes behavioural patterns and information flow, while infectious diseases continue to spread primarily through human movement and physical contact. In this review, we have highlighted the separate roles of these networks on infectious disease dynamics, summarised existing modelling strategies, and identified key research directions to bridge existing knowledge gaps.

### Integrating social influence into population-level models

Population-level modelling, such as compartmental and meta-population models, remains an essential tool in the rapid assessment of infectious disease spreading. However, despite growing studies that have underscored the mutual impacts of social and behavioural factors on disease transmission, most models overlook the interdependence of individual behaviours, which are largely modulated by social networks. These interactions are crucial for understanding behavioural cascades such as vaccine acceptance or risk avoidance. Therefore, we recommend a two-step approach for future work: first identify collective behavioural shifts induced by social interactions, and then incorporate these dynamics into transmission models, potentially through a network-based behavioural adjustment parameter influencing infection rate.

### Leveraging GABM and multiplex networks for individual-level modelling

We highlight GABM and multiplex network analyses as promising modelling approaches for capturing behavioural heterogeneity and co-evolving contagion dynamics at the individual level. GABM, powered by large language models, allows agents to perceive and interpret the social environment dynamically and make autonomous decisions, such as whether to vaccinate or comply with public health policies based on evolving social norms and personal beliefs. These models have shown promise across domains, including urban planning, transportation, and epidemic forecasting. Multiplex networks offer another promising structural framework for modelling cross-layer interactions, such as how awareness spreads in a social network and alters behaviour in a mobility and contact network. This enables simulation of co-evolutionary dynamics between information dissemination and infectious disease spreading. Moreover, multiplex networks can explicitly accommodate heterogeneous individual awareness, peer influence, and context-dependent behaviour through both within-network interactions and cross-network dynamics, enabling contagion effects in the information communication layer to be cascaded to influence disease dynamics.

Recent advances in large language models also offer promising capabilities for developing multimodal, self-aware, autonomous multiplex disease models, in which each layer (e.g., mobility, contact, communication) can be designed analogously to GABM. However, such models remain largely simulation-based, constrained by the lack of comprehensive, individual-level data records across all network layers. Although such granular and complete datasets are rarely available in reality, emerging data sources may partially address this challenge.

### Incorporating emerging data sources and robust inference for integrated models

Novel data streams offer unprecedented opportunities to understand the joint influences of human mobility and social interactions. For example, the massive volume of social media data, despite its demographic biases, enable simultaneous extraction of online social networks and mobility patterns from geotagged posts, facilitating the construction of unified online and offline human interaction networks. Future research should leverage multi-source data to integrate information dissemination, behavioural responses, and physical contact, thereby quantifying comprehensive infectious disease mechanisms across space and time. Although comprehensive data collection is essential for constructing social networks and modelling infectious diseases, this must be balanced against the need for timely outbreak responses, resource constraints, social costs, and privacy concerns. Researchers should adapt data collection and integration strategies, depending on intervention goals, data infrastructure, human resources, and epidemiological contexts [158], while safeguarding individual confidentiality under ethical approvals and data protection policies across different jurisdictions.

In addition, a critical limitation of existing studies is the reliance on cross-sectional or correlational analyses, which constrains the ability to infer causal relationships, and therefore, the estimated effectiveness of public health policies informed by network studies. Future work should consider model validation using data obtained from more rigorous designs, such as longitudinal cohort studies, randomised controlled trials, and quasi-experimental approaches where appropriate, to better assess how social, mobility and contact networks influence behavioural adoption and disease transmission. Such methodologically robust approaches and causal evidence are essential for guiding targeted and precise public health interventions.

### Addressing demographic disparities and post-epidemic social reintegration

Demographic characteristics, such as age, gender, race, and socioeconomic status, substantially influence social mixing patterns, risk exposure, and access to interventions, which remain understudied for infectious diseases beyond STIs. For example, the COVID-19 pandemic exemplified age-related disparities, with younger peers showing higher risks of mutual infection during early transmission [159] and older adults experiencing increased social marginalization due to greater clinical severity [160]. Such inequalities are expected to sustain over a long period post COVID-19 [161]. Future modelling efforts should explicitly incorporate demographic structure and inequities, both in the formation of social, mobility and contact networks and in differential behavioural responses. This is essential for informing equitable public health strategies and ensuring that marginalised populations receive timely, appropriate support.

In addition, while preventive behaviours and interventions such as vaccination and PrEP have received substantial attention in existing studies, therapeutic norms and recovery behaviours remain underexplored [162]. Research should examine how social networks shape post-illness or post-epidemic behaviours, including medical treatment adherence and social reintegration [163]. Understanding these behaviours spread through social ties can inform strategies for socioeconomic resilience and long-term health system recovery following epidemics or pandemics. By addressing both demographic disparities and post-epidemic reintegration in network-informed modelling, we can better understand the full lifecycle of epidemic impact and design more holistic public health strategies.

## Conclusions

As the world grows more socially, digitally, and physically interconnected, the task of modelling and intervening in infectious disease dynamics becomes increasingly complex. Understanding how diseases spread is no longer just a matter of tracking pathogens through mobility and contact patterns; it also requires attention to how human behaviour evolves, how people influence each other, and how information moves through networks. This review has shown that while we have made substantial progress in modelling mobility- and contact-driven transmission, the integration of social, behavioural, and information diffusion processes into infectious disease models remains insufficient. Some approaches can explicitly integrate social networks into mobility-driven disease models, including multiplex network analyses based on structural similarities of different networks, and also generative agent-based models taking advantage of large language models. However, these approaches remain largely theoretical and confined to simulations, due to the scarcity of high-resolution, multi-source data across social, spatial, and behavioural dimensions.

Therefore, as we look ahead, it is vital to prioritise efforts that bridge data and model complexity, and also develop scalable methods that practically introduce social network influences onto disease transmission across varied contexts and broader, vulnerable populations. This means integrating diverse datasets, such as geospatial movement, digital communication and connections, and health behaviours, while also being mindful of privacy, ethical concerns, and the need for rapid, real-time insights. It is encouraged that the governments coordinate public health policies, curb the spread of misinformation, and reallocate resources to socially marginalised groups. A multidisciplinary, cross-sector, and inclusive approach would strengthen the research and inform more targeted intervention strategies for infectious disease control.

## Supplementary Information

Supplementary Material 1: List 3 for literature exclusion: ageing, aging, animal, brain, brand, business, cancer, chem*, chronic, commercial, computer, consume*, corporate, crimin*, diabet*, ecolog*, econo*, educat*, energy, engineering, financ*, firm, food, indust*, insect*, justice, land cover, land use, library, livestock, market*, maturitas, mice, midwifery, monetary, neuro*, nurse*, nursing, nutrition, ornitholog*, orthodontics, paediat*, parasitolog*, patholog*, pharmac*, polit*, project, psychia*, psycho*, raccoon*, rangeland, rat, shakehold*, sociolog*, species, stock, suicide, technolog*, touris*, tropical.

## Data Availability

The publication metadata were collected from Web-of-Science Core Collection (WoSCC) and PubMed. For WoSCC, we explored two sub-datasets: Science Citation Index Expanded (SCIE) and Social Science Citation Index (SSCI). Data from WoSCC are not publicly available and were used under license from their respective publishers. PubMed is publicly available (https://ftp.ncbi.nlm.nih.gov/pubmed/).

## Abbreviations

ABMs: Agent-based models
CDR: Call detail record
COVID-19: Coronavirus disease 2019
GABM: Generative agent-based modelling
GPS: Global positioning system
HIV: Human immunodeficiency virus
LLMs: Large language models
nCov: Novel coronavirus
NHS: National Health Service
PrEP: Pre-exposure prophylaxis
PWID: People who inject drugs
RCT: Randomized controlled trial
SARS: Severe acute respiratory syndrome
SARS-CoV-2: Severe acute respiratory syndrome coronavirus 2
STIs: Sexually transmitted infections
VBDs: Vector-borne diseases
