# Supplementary material for: Social, mobility and contact networks in shaping health behaviours and infectious disease dynamics: a scoping review
Source: Infect Dis Poverty. 2025 Dec 3;14:123. doi: 10.1186/s40249-025-01378-6 (PMC12673763; doi:10.1186/s40249-025-01378-6)
Supplement: Supplementary file 1 — Supplementary Material 1: List 3 for literature exclusion: ageing, aging, animal, brain, brand, business, cancer, chem*, chronic, commercial, computer, consume*, corporate, crimin*, diabet*, ecolog*, econo*, educat*, energy, engineering, financ*, firm, food, indust*, insect*, justice, land cover, land use, library, livestock, market*, maturitas, mice, midwifery, monetary, neuro*, nurse*, nursing, nutrition, ornitholog*, orthodontics, paediat*, parasitolog*, patholog*, pharmac*, polit*, project, psychia*, psycho*, raccoon*, rangeland, rat, shakehold*, sociolog*, species, stock, suicide, technolog*, touris*, tropical. [file 40249_2025_1378_MOESM1_ESM.pdf]

Social, mobility, and contact networks in shaping health behaviours and infectious disease dynamics: a scoping review

—— reply to reviewers' comments

We are grateful for the careful reviews and constructive suggestions raised by three reviewers. We have made substantial attempts to address all the comments in this updated manuscript and believe our manuscript has been remarkably improved.

Please find below our point-by-point responses to the reviewers' suggestions and concerns. Their original comments are in *italic* and highlighted in *blue*, and our responses are in regular font. We mark all the changes in purple in the revised manuscript.

Reviewer #1: This scoping review provides a comprehensive synthesis of the roles of social, mobility, and contact networks in infectious disease dynamics, highlighting their interconnectedness and gaps in current research. The manuscript is well-structured, methodologically rigorous, and addresses a timely topic. However, several major and minor revisions are needed to enhance clarity, depth, and applicability.

1.The review discusses social, mobility, and contact networks but lacks a unified theoretical framework to integrate these concepts. A dedicated section or diagram synthesizing how these networks interact (e.g., feedback loops, synergistic effects) would strengthen the conceptual contribution.

Response [1]:

Thank you for these valuable suggestions. Reviewer #3 raises similar concerns in his/her Comments [3-5]. We acknowledge that our previous review on integrating social, mobility and contact networks into disease modelling was relatively limited. In the revised manuscript, we have substantially expanded the final chapter "Epidemiological models integrating social, mobility and contact networks". We introduced the evolution of commonly used disease modelling strategies, ranging from compartmental and metapopulation models, to agent-based and multilayer models, and finally AI-enabled stochastic modelling (your Comment [6]). In accordance with your advice, we specifically cited references in Economic Epidemiology to introduce the feedback loops between human behaviours and disease prevalence, emphasizing the importance of heterogenous individual behaviours in shaping disease dynamics.

The chapter is now well organised, covering the range from population-level modelling to individual-level studies; from empirical analysis with real data to simulation studies; and from separate integration of behavioural dimensions to disease-behaviour co-evolution. We hope that this will provide a better conceptual framework than before.

We copy the outline of our revised chapter here for your reference, please kindly refer to lines 400-559 in the manuscript for full revisions:

> **Epidemiological models integrating social, mobility and contact networks**
>
> ***Population-level modelling with mobility and contact networks***
>
> Modelling strategies largely determine the extent to which and how well we can translate knowledge of social, mobility and contact networks into more accurate estimation and prediction of infectious disease dynamics. Traditional population-level models…
>
> ***Incorporating behavioural feedback and social influences***
>
> Beyond mobility, social and behavioural factors are also worth being incorporated into disease modelling…
>
> ***Individual-level modelling: coupling social, mobility, and contact networks***
>
> Theoretical advances and emerging computational techniques demonstrate the potential of integrating intrinsically linked social factors into infectious disease models, where one key

amendment is to shift the modelling strategy from “top-down” (population-level) to “bottom-up” (individual-level) …

Agent-based models (ABMs) usually serve as a basic choice for “bottom-up” modelling…

Multilayer and multiplex networks in ABMs

One key extension of AMBs involves embedding agents within multilayer or multiplex networks to more realistically simulate interactions across physical, social and informational domains…

Stochastic agent-based models and generative agents

A further evolution of traditional rule-based ABMs involves the incorporation of stochastic processes, allowing models to account for the randomness and variability of human behaviours…

The recent advent of large language models (LLMs) has provided further opportunities for creating generative agents – autonomous, language-driven individuals capable of making context-aware decisions based on evolving information…

From simulation to real-world application

Although ABMs have long been a proven approach for disease transmission modelling, their variants explicitly introducing individual awareness and social network impacts, including multiplex network and GABM, remain primarily simulation-based …

2.The review summarizes network-driven interventions (e.g., targeting super-spreaders) but lacks a systematic evaluation of their efficacy across contexts. A table comparing intervention outcomes (e.g., vaccination uptake, mobility reduction) by network type would be valuable.

Response [2]:

We appreciate your thoughtful suggestion regarding a comparative table on the efficacy of network-driven interventions. While we agree that such a synthesis would be valuable in principle, we believe it is not applicable within the scope of the present work.

This manuscript is a scoping review, not a systematic review or meta-analysis. The included literature spans multiple research areas rather than focusing on a single specific domain. We included only the papers that contribute the most to our storyline, which means that the literature from each research area is far from comprehensive. Therefore, we believe that a table summarising the intervention efficacy of several representative studies might not constitute a reliable and robust comparison.

For example, Laranjo et al. (2015) summarise the effect of social network interventions on health behaviour changes. Although our reference has a few overlaps with theirs, we don’t think a comparison without a systematic literature search is as valuable as their review. The same is true for other network-driven interventions (such as targeting super-spreaders, as you mentioned), which is again just one area of research covered in this review, not its sole primary focus.

Ref: Laranjo, L. et al. The influence of social networking sites on health behavior change: a systematic review and meta-analysis. J. Am. Med. Inform. Assoc. 22, 243–256 (2015).

Ethical and Privacy Concerns.

3.Add a column to Table 1 listing representative studies for each network type to enhance reproducibility.

Response [3]:

Great suggestion! We now add one column of representative studies as you suggested. In particular, we briefly describe the research questions rather than simply citing them.

Table 1. Strengths, limitations and examples of social, mobility and contact networks for health behaviours and infectious diseases modelling

Network type and models		Strengths	Limitations	Example studies
Social networks	Regression models	• Direct relationship between individuals' and friends' behaviours<br>• Longitudinal cohort study can reveal causality	○ Surveys are costly, limited to small samples, and potentially biased to the general population<br>○ Cross-sectional design makes strong causal inference challenging when rounds of survey are limited	Having more friends who smoke increases the probability of smoking [16]<br>Alcohol consumption is clustered and socially contagious in social networks [56]
	Network models	• Explicit dynamics of information and behaviour contagion process within social networks<br>• Feasible for designing targeted network intervention	○ Real-world validation needs rigorous pre-experimental design, e.g., randomized controlled trial (RCT)<br>○ Simulations and synthetic samples are widely used when real data are unavailable or static	Promoting running programs through a Facebook-based social network intervention [20]<br>Interventions via social networks help reduce population obesity [74]<br>Bots increase exposure to negative and inflammatory contents online [48]
Mobility and contact networks	Compartmental / metapopulation models	• Simple framework allows rapid assessment of disease transmission<br>• Low data requirement with no need of human activity records, or rely on human mobility across regions only	○ Homogeneous mixing assumption deviates from reality<br>○ Individual behavioural discrepancies are mostly ignored	Spread and dynamics of the COVID-19 epidemic in China [30] and Italy [129]<br>Predict cholera outbreak in Haiti across communities [130]
	Agent-based models (ABMs)	• Individual-level modelling enables various behaviours across individuals to be included<br>• Reflect real-world complexities	○ High in data requirements, computing resources and output uncertainty	ABM evaluates COVID-19 transmission [137]<br>Effect of post-lockdown measures on severe COVID-19 infection in France [161]
Integrated social, mobility and contact networks	Multiplex network-based models	• Explicitly connect spreading processes of information, behaviours, and diseases<br>• Flexible, multi-layer structures can be expanded to better resemble real-world complexities	○ Detailed individual-level data on both social and physical interactions is hard to obtain<br>○ Primarily simulation-based, with limited real-world application	The interplay between three-layer of awareness, behaviours, and epidemics [152]<br>The impact of node activities on virus spreading [153]
	Generative agent-based models (GABMs)	• Generative agent acts resembling human thoughts and behaviours in the real world<br>• Accept information directly instead of predefined rules<br>• Allow dynamic adjustment	○ Challenging in model explainability and reproducibility	Connect each agent to generative large language model so that generative agents can mimic real-world behaviours [162]

4.Revise awkward phrasing (e.g., "Scholars need to make trade-offs..." → "Researchers must balance model complexity with data availability"

Response [4]:

Thanks for pointing out this issue. The sentence you mentioned has now been deleted and replaced by other content in the revised manuscript. Nonetheless, we have reviewed the manuscript and have avoided any other awkward phrasing.

5. can add the reference of long COVID19 with the social connection in the fourth content of your discussion, such Long COVID: Current Status, Challenges and Future Directions. (2024). ICell, 1(1). https://doi.org/10.71373/ELJT3204.

Response [5]:

Thanks for your advice. We have now incorporated the paper you suggested into our discussion.

> … the COVID-19 pandemic exemplified age-related disparities, with younger peers showing higher risks of mutual infection during early transmission[172] and older adults experiencing increased social marginalization due to greater clinical severity[173]. Such inequalities are expected to sustain over a long period post COVID-19[174].
>
> [174] Huang, L. et al. Long COVID: Current Status, Challenges and Future Directions. *iCell* 1, (2024).

6. Include 2024-2025 studies on AI-driven network modeling (e.g., LLMs for behavior prediction) to reflect cutting-edge advances.

Response [6]:

Thanks for pointing out this important point. We admit that we missed recent publications because our previous literature search ended at the end of 2023. Following your advice, we supplemented recent studies on AI-driven network modelling. Instead of LLMs for behaviour prediction, we discussed LLMs for generative agent-based modelling, which matches better with our storyline. You may have already seen our revisions in the Response [1].

> … The recent advent of large language models (LLMs) has provided further opportunities for creating generative agents – autonomous, language-driven individuals capable of making context-aware decisions based on evolving information[164]. Rather than determining certain assumptions about what behavioural dimensions to include and how each human would act, generative agent-based modelling (GABM) accepts diverse information directly and each agent makes their own decision-making on behavioural changes based on existing knowledge and the social environment, which can be defined using user prompt and system prompt, respectively[165, 166]. More importantly, researchers can pass new information (e.g., public health messages, news updates, or peer behaviours) to the agent at each time step, enabling dynamic responses that more closely resemble real-world behaviour over time. By combining stochastic behavioural diversity with natural language processing capabilities, GABMs offer a powerful tool for exploring the cognitive and social dimensions of epidemics, especially in complex, dynamic environments where traditional rule-based approaches fall short.

7.Spell out "VBDs" and "STIs" at first use (currently undefined in Abstract).

Response [7]:

Thank for emphasizing this point. We have spelled out “VBDs” and “STIs” when they first appear in the main text.

> … Social networks can similarly shape the dynamics of vector-borne diseases (VBDs) such as malaria...

… In sexually transmitted infections (STIs), social networks significantly influence transmission risk…

8. The conclusion reiterates findings without emphasizing policy implications. Highlight 1-2 actionable insights for public health practitioners.

Response [8]:

Thank you for your suggestions. We have now added a few final policy implications at the end of the *Conclusion*

… This means integrating diverse datasets, such as geospatial movement, digital communication and connections, and health behaviours, while also being mindful of privacy, ethical concerns, and the need for rapid, real-time insights. It is encouraged that the governments coordinate public health policies, curb the spread of misinformation, and reallocate resources to socially marginalised groups. A multidisciplinary, cross-sector and inclusive approach would strengthen the research and inform more targeted intervention strategies for infectious disease control.

Again, we thank the reviewers for their suggestions and great help with our manuscript.

Reviewer #2: This study on the review of "Social, mobility, and contact networks in shaping health behaviors and infectious disease dynamics" assesses current research on the interaction between social networks, mobility, and contact in the transmission of infectious diseases using several search engines. It aims to enhance the effectiveness of using social networks, mobility, and contact in the dynamics of infectious diseases. Through a detailed analysis of several search engines, the study highlighted the necessity of incorporating behavioral, informational, and epidemiological processes into disease models that are currently disparate.
This incorporation is even more useful as human mobility and physical contact networks determine the spatial-temporal transmission of pathogens, as demonstrated by recent pandemics of Ebola, dengue, and COVID-19, etc.
The scope of this review is that it discusses advances in the impact of networks, mobility, and contact on the formation of health behaviors and the dynamics of infectious diseases. Moreover, it aims to identify critical gaps in knowledge and research on infectious disease modeling and its applications. Finally, to strengthen public health strategies, allowing for equitable and effective interventions against emerging infections, the authors propose to prioritize the integration of multi-source data, the unification of models, the exploration of demographic inequalities, and the implementation of robust causal inference designs.
In summary, the study caught my attention and deserves to be published.

Response:

Thank you for your comments on our manuscript. We appreciate that you recognised the value of our work. In the revised manuscript, we made further improvements to it:

1. We revised the *Methods* chapter by optimizing the literature searching and screening process. Figure 1 has also been updated to a more professional style.
2. We substantially expanded the final chapter "Epidemiological models integrating social, mobility and contact networks" (lines 400-559). We introduced the evolution of commonly used disease modelling strategies, ranging from compartmental and metapopulation models, to agent-based and multilayer models, and finally AI-enabled stochastic modelling. The chapter now provides a more comprehensive methodological framework than before, covering the range from population-level modelling to individual-level studies; from empirical analysis with real data to simulation studies; and from separate integration of behavioural dimensions to disease-behaviour co-evolution.
3. The table has been redesigned to reflect the revised chapter. It now lists strengths and limitations of different types of network study, together with an additional column of representative studies using each approach.

Reviewer #3: Current research lacks a comprehensive synthesis of how information dissemination, behavioral contagion and pathogen transmission interdependently influence disease dynamics. This review aims to bridge these gaps by synthesizing current research on the roles of social, mobility and contact networks in infectious disease transmission. How to synthesize current research on the interplay between social and contact networks in infectious disease transmission is important. The current study provides many examples, but does not distill them into key insights or findings as expected from a scoping review. A more structured synthesis of the literature and clearer identification of patterns or gaps would enhance its value to the field. A significant limitation of the manuscript is the lack of clarity in how the final studies were included. Here I will give some suggestions and comments that I hope can improve the quality of the study.

1. I couldn't find the time frame during which the literature search was conducted.

Response [1]:

Thanks for pointing out this issue. This revised manuscript now includes literature searched from January 2000 to June 2025.

Previously, the literature search was conducted by the end of 2023, which means that recent publications were omitted. However, we notice that your Comment [5] and the Comment [6] from Reviewer #1 both point out that studies from 2024 to 2025, especially those adopting emerging techniques like AI, are missing. Therefore, we supplemented the literature records. Figure 1 (flowchart) and Figure 2 (keywords co-occurrence network) were also updated accordingly. Please refer to Response [2] for details.

2. The final inclusion/exclusion step is not described. This is a serious weakness that I think should be improved. The review reports identified 9,112 records and ultimately included 79 studies (i.e. fewer than 1% were selected), but the rationale for this substantial reduction is not made transparent. There is little explanation of the inclusion and exclusion criteria applied during this step. For a scoping review, greater transparency is essential, including a detailed breakdown of exclusion reasons, which is required in this study. Without clear criteria, the authors might just pick articles they wanted. A PRISMA-style flow diagram and clearer criteria would strengthen reproducibility and clarity. It is essential to provide a reproducible flow of how studies were filtered. Justification for why many relevant-sounding studies (e.g., on social contagion modeling) were excluded should be given. For example, PMID: 21079667 and PMID: 34812363, which provide valuable insights related to infectious disease modelling and social contagion, were missing.

Response [2]:

Thank you very much for pointing out this major flaw. We acknowledge that shrinking the literature size to 1% at one step could be confusing to readers and would reduce the reliability of our analysis. In the revised manuscript, we clarified that the final-step inclusion criterion was to search for papers related to infectious disease spreading or social network influences across all those about public health applications.

As you suggested, we have also updated Figure 1 using PRISMA-style.

> … Finally, we kept papers potentially relevant to infectious disease spreading or social network influences by searching their titles and abstracts with keywords (List 4): spread*, transmission,

transmit*, peer influence, peer effect, network effect, network intervention. The four steps combined retains 1,590 records (Fig. 1). After reading and manual selection, 73 publications were finally included in this review.

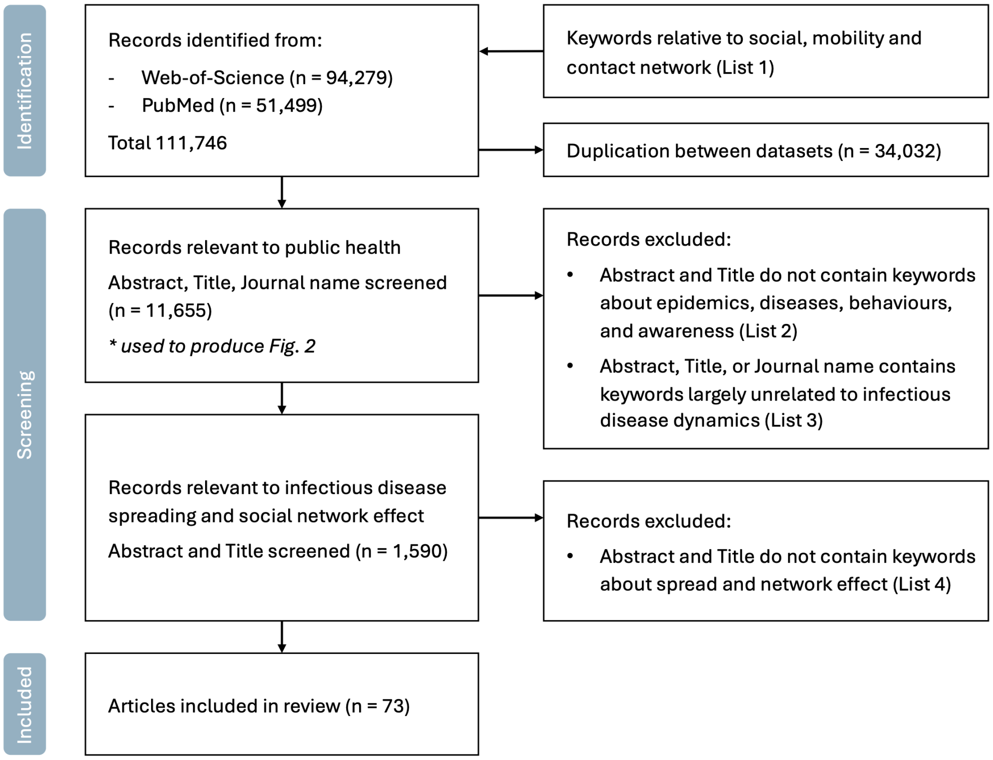

Figure 1. Flowchart of literature searching and selection.

We justify our previous methods from two fronts. First, the intermediate step reported thousands of records, which seems a lot. One reason for maintaining such a large set of candidate literature is that our scoping review does not focus on only one scope, which is why we were able to generate a keywords co-occurrence network across multiple fields (network dynamics, individual behaviours, health risks, and information dissemination, Figure 2).

Second, we acknowledge that we previously used suboptimal methods in the literature screening process. In particular, we set overly stringent criteria for filtering journal names. For example, we excluded all IEEE journals, which are mostly in the field of Electrical, Electronics, and Engineering, except for only a few interdisciplinary journals. This is why we overlooked the IEEE Access paper you mentioned (PMID: 34812363). The revised manuscript has relaxed this criterion to include more eligible papers.

3. In this scoping review, I will expect the authors to provide two important information from the papers. One is what type of network study has been used (especially in recent years), and the other is what data sources are currently available for the network study. In addition, authors should help to bridge the learning gap for public health readers to understand how to use network analysis or to communicate with modelers.

Response [3]:

Thank you for these two suggestions. We are sorry that we feel a bit unclear about what you meant by "what type of network study has been used". By referring to your next comment, we assume you are asking about different approaches / modelling strategies used in network studies to model disease dynamics. We have substantially revised the relevant chapters in the revised manuscript, and we hope this could provide useful information for researchers and public health practitioners to understand and use network analysis. Please kindly refer to the next Response [4] for details.

Regarding your next point about data, we have now added a paragraph in the Introduction that briefly discusses data commonly used in network studies.

> … Fortunately, increasing availability and variability of diverse human activity footprint data enable a comprehensive understanding of complex social interactions for infectious disease dynamics. On the one hand, traditional disease models solely based on mobility and contact networks already have a rich selection of data sources. Those include records from cellular signalling[32–34], GPS positioning[35], and public transportation[2, 3], enabling investigation of disease transmission risks attributed to multi-scale human mobility from intra-city, inter-city, to international[1]. Further, new data sources provide opportunities to measure multi-dimensional information simultaneously, especially those related to societal and social interactions in addition to mobility and contact. For instance, mobile phone data[36] and geotagged social media data[37] can be used to infer both human mobility patterns and friendship network structure. Mobile health code records, like data from the NHS COVID-19 app, can reflect both close physical contact and social relationships[38].

4. The definition or classification of each network study is unclear. Table 1 provides different types of networks. However, how the reviewed articles belonged to different categories is unknown in this study. For example, despite the collection of studies using large social networks (e.g. smoking in social networks). The study should have an additional table or use some way to list all the network studies and then classify them. In evaluating models that integrate epidemic and social contagion dynamics, some key features are important to distinguish. For example, the authors can consider disease-behavior co-evolution, data fitting, joint parameter estimation and statistical approach. First, disease-behavior co-evolution refers to whether the model explicitly allows the disease dynamics (e.g., infection spread) and behavioral responses (e.g., awareness, protective actions) to influence each other over time, often modeled via coupled equations or multilayer networks. Second, empirical data fitting asks whether the model parameters are calibrated using real-world data—such as case counts, mobility patterns, or survey responses—to make the model predictive or interpretable in specific contexts. Finally, joint parameter estimation refers to whether parameters for both the disease process and the behavioral process are estimated simultaneously, rather than assuming one set (e.g., behavior) is known or fixed. This joint estimation is often more challenging, as behavioral dynamics are typically less observable and more uncertain than epidemiological ones, yet it is crucial for capturing feedback effects and making reliable inferences early in an outbreak. I assumed there would be statistical-based approach to allow certain relationships to be determined.

Response [4]:

Thank you for this very thoughtful comment, we understand your concerns about the definition and classification of each network type. We made several revisions to make it much clearer.

First, as you correctly understood in Comment [8], this review mainly involves two major network types: 1) *social network* and 2) *mobility and contact network*, where social network refers to social interactions facilitating information diffusion and behaviour adoption, and mobility and contact network is more related to human movement. To avoid confusion, we now define them at the very beginning in the main text and avoid using short names (previously *mobility and contact networks* was shortened as contact networks, which might confuse you). Please refer to Response [8] for details about this.

> … Importantly, human mobility and contact networks (*mobility and contact networks* hereafter), a particular form of geographical network summarising human movement and physical contact, fundamentally shape patterns of infectious disease transmission[1]. As human hosts travel and interact physically, pathogens can be carried and transmitted…

> … social interactions and personal relationships (referred to as *social networks*)[11–13]. Within social networks, ties between people, whether through in-person or online interactions, enable knowledge, awareness, and cognition to spread across individual, local and global scales…

Second, your ideas about features to distinguish models integrating epidemic and social contagion dynamics are quite valuable. We realise that previously the transition between the first two paragraphs in the chapter about integrated models was a bit abrupt, especially when the concept of multiplex network was not introduced earlier (your Comment [5]). In the revised manuscript, we enriched the content of the relevant chapter to introduce the development of commonly used disease modelling strategies, ranging from compartmental and metapopulation models, to agent-based and multilayer models, and finally AI-enabled stochastic modelling. The chapter is now reorganised according to key features you suggested: from population-level modelling to individual-level modelling; from empirical analysis using real data to simulation study; and from separate integration of behavioural dimensions to disease-behaviour co-evolution.

We copy the outline of our revised chapter here for your reference, please kindly refer to lines 400-559 in the manuscript for full revisions:

> **Epidemiological models integrating social, mobility and contact networks**
>
> ***Population-level modelling with mobility and contact networks***
>
> Modelling strategies largely determine the extent to which and how well we can translate knowledge of social, mobility and contact networks into more accurate estimation and prediction of infectious disease dynamics. Traditional population-level models…
>
> ***Incorporating behavioural feedback and social influences***
>
> Beyond mobility, social and behavioural factors are also worth being incorporated into disease modelling…
>
> ***Individual-level modelling: coupling social, mobility, and contact networks***
>
> Theoretical advances and emerging computational techniques demonstrate the potential of integrating intrinsically linked social factors into infectious disease models, where one key

amendment is to shift the modelling strategy from “top-down” (population-level) to “bottom-up” (individual-level) …

Agent-based models (ABMs) usually serve as a basic choice for “bottom-up” modelling…

Multilayer and multiplex networks in ABMs

One key extension of AMBs involves embedding agents within multilayer or multiplex networks to more realistically simulate interactions across physical, social and informational domains…

Stochastic agent-based models and generative agents

A further evolution of traditional rule-based ABMs involves the incorporation of stochastic processes, allowing models to account for the randomness and variability of human behaviours…

The recent advent of large language models (LLMs) has provided further opportunities for creating generative agents – autonomous, language-driven individuals capable of making context-aware decisions based on evolving information…

From simulation to real-world application

Although ABMs have long been a proven approach for disease transmission modelling, their variants explicitly introducing individual awareness and social network impacts, including multiplex network and GABM, remain primarily simulation-based …

We have to clarify that we did not mean to ignore other modelling approaches besides multiplex analysis. Instead, the fact is that studies incorporating social network impacts into disease modelling using real-world data are extremely scarce, which is exactly the major research gap identified by this review, rather than its shortcoming. In our revision, although we have reviewed different methods for incorporating social factors into disease modelling, they still primarily focus on “social impact” rather than “social network impact”. It is the area where future research can delve deeper. We also provide our thoughts about this in the *Discussion*, as you suggested in Comments [7]. Please refer to Response [7] for details.

Third, Reviewer #1 raised the same concern that typical references should be linked with the corresponding network types. In the revised manuscript, we updated Table 1 by listing representative studies in an additional column. In particular, we briefly describe the research questions rather than simply citing them.

Table 1. Strengths, limitations and examples of social, mobility and contact networks for health behaviours and infectious diseases modelling

Network type and models		Strengths	Limitations	Example studies
Social networks	Regression models	• Direct relationship between individuals' and friends' behaviours<br>• Longitudinal cohort study can reveal causality	○ Surveys are costly, limited to small samples, and potentially biased to the general population<br>○ Cross-sectional design makes strong causal inference challenging when rounds of survey are limited	Having more friends who smoke increases the probability of smoking [16]<br>Alcohol consumption is clustered and socially contagious in social networks [56]
	Network models	• Explicit dynamics of information and behaviour contagion process within social networks<br>• Feasible for designing targeted network intervention	○ Real-world validation needs rigorous pre-experimental design, e.g., randomized controlled trial (RCT)<br>○ Simulations and synthetic samples are widely used when real data are unavailable or static	Promoting running programs through a Facebook-based social network intervention [20]<br>Interventions via social networks help reduce population obesity [74]<br>Bots increase exposure to negative and inflammatory contents online [48]
Mobility and contact networks	Compartmental / metapopulation models	• Simple framework allows rapid assessment of disease transmission<br>• Low data requirement with no need of human activity records, or rely on human mobility across regions only	○ Homogeneous mixing assumption deviates from reality<br>○ Individual behavioural discrepancies are mostly ignored	Spread and dynamics of the COVID-19 epidemic in China [30] and Italy [129]<br>Predict cholera outbreak in Haiti across communities [130]
	Agent-based models (ABMs)	• Individual-level modelling enables various behaviours across individuals to be included<br>• Reflect real-world complexities	○ High in data requirements, computing resources and output uncertainty	ABM evaluates COVID-19 transmission [137]<br>Effect of post-lockdown measures on severe COVID-19 infection in France [161]
Integrated social, mobility and contact networks	Multiplex network-based models	• Explicitly connect spreading processes of information, behaviours, and diseases<br>• Flexible, multi-layer structures can be expanded to better resemble real-world complexities	○ Detailed individual-level data on both social and physical interactions is hard to obtain<br>○ Primarily simulation-based, with limited real-world application	The interplay between three-layer of awareness, behaviours, and epidemics [152]<br>The impact of node activities on virus spreading [153]
	Generative agent-based models (GABMs)	• Generative agent acts resembling human thoughts and behaviours in the real world<br>• Accept information directly instead of predefined rules<br>• Allow dynamic adjustment	○ Challenging in model explainability and reproducibility	Connect each agent to generative large language model so that generative agents can mimic real-world behaviours [162]

We noticed that you wish us to list all the network studies of each category. However, we would like to express our concerns that enumeration is not the priority of this review. This manuscript is a scoping review instead of a systematic review. The included literature involves multiple study areas rather than focusing on one specific domain. It means that we have to make a trade-off between all the existing literature and the included articles. Our goal was to select those that made the greatest contributions to form our storyline, rather than to select and cite all the articles that fell within the scope of our review.

5. Although the study cited a few important network analysis papers, many of the important works were published more than 10 years ago. The review seems a little bit too shallow and overly brief. The section Modelling the interplay between social, mobility and contact networks on infectious diseases addresses a crucial area, but its current content can be origanized in a better way. The author introduced multiplex network analysis in the second paragraph of this section and described it in detail throughout the manuscript. However, the concept and implementation of multiplex networks are not new. I suggest the authors move part of this section into the Introduction and provide an earlier framing of multiplex network modeling.

Response [5]:

Thanks for pointing out this issue. We admit that our citation disproportionally concentrates on classic literature while leaving new methods underrepresented. This drawback could be pronounced particularly in this age where artificial intelligence explosively developed. Reviewer #1 also raised this concern in

his/her Comment [6]. In the revised manuscript, we cited more recent work and particularly those relevant to AI when introducing modelling strategies.

> … The recent advent of large language models (LLMs) has provided further opportunities for creating generative agents – autonomous, language-driven individuals capable of making context-aware decisions based on evolving information[164]. Rather than determining certain assumptions about what behavioural dimensions to include and how each human would act, generative agent-based modelling (GABM) accepts diverse information directly and each agent makes their own decision-making on behavioural changes based on existing knowledge and the social environment, which can be defined using user prompt and system prompt, respectively[165, 166]. More importantly, researchers can pass new information (e.g., public health messages, news updates, or peer behaviours) to the agent at each time step, enabling dynamic responses that more closely resemble real-world behaviour over time. By combining stochastic behavioural diversity with natural language processing capabilities, GABMs offer a powerful tool for exploring the cognitive and social dimensions of epidemics, especially in complex, dynamic environments where traditional rule-based approaches fall short.

Regarding your concern about a better way to organize the section on integrated modelling, you may have already seen our revisions in the previous Response [4].

Based on the current revision, we thought the concept of multiplex network is now well placed in the methodology framework. It is now simply one of the suggested methods instead of the only output as previously. Therefore, it no longer warrants mentioning it upfront in the *Introduction*. We prefer to keep it as is. If you still think early framing is important, please let us know and we will be happy to revise it.

6. On line 499, "Second, emerging data sources offer unprecedented opportunities to understand the joint influences of human mobility and social interactions." Data sources should be cited. In addition, the studies that provide important data sources should be listed in the main text before the discussion.

Response [6]:

Thanks for your comments. The Comment [3] raises the same issue. You may have seen in the Response [3] that we added a paragraph in the *Introduction* describing data sources with key reference cited.

7. How homophily and mobility can be used to calibrate or influence social networks is lacking. What is the authors' perspective on integrating social and contact networks into infectious disease modeling using real-world data? Should detailed frameworks such as agent-based models be used, or can compartmental models be useful? Do the authors recommend simpler approaches, such as mean-field approximations, for parameter estimation based on empirical data? Clarifying this would help readers understand the practical modeling strategies suitable for real-world applications.

Response [7]:

Thank you for this thoughtful comment. We noticed that these points are mostly relevant to your previous Comment [4], and you can find in our previous response that we have reviewed and summarised the different modelling strategies and data use in the revised manuscript.

Following those revisions, we followed your advice here and enriched the *Discussion* by providing our thoughts.

> … In this review, we have highlighted the separate roles of these networks on infectious disease dynamics, summarised existing modelling strategies, and identified key research directions to bridge existing knowledge gaps.
>
> ***Integrating social influence into population-level models***. Population-level modelling such as compartmental and meta-population models remain essential tools in the rapid assessment of infectious disease spreading. However, despite growing studies have underscored the mutual impacts of social and behavioural factors on disease transmission, most models overlook the interdependence of individual behaviours, which are largely modulated by social networks. These interactions are crucial for understanding behavioural cascades such as vaccine acceptance or risk avoidance. Therefore, we recommend a two-step approach for future work: first identify collective behavioural shifts induced by social interactions, and then incorporate these dynamics into transmission models, potentially through a network-based behavioural adjustment parameter influencing infection rate.
>
> ***Leveraging GABM and multiplex networks for individual-level modelling***. We highlight GABM and multiplex network analyses as promising modelling approaches for capturing behavioural heterogeneity and co-evolving contagion dynamics at individual-level. GABM, powered by large language models, allows agents to perceive and interpret the social environment dynamically and make autonomous decisions, such as whether to vaccinate or comply with public health policies based on evolving social norms and personal beliefs. These models have shown promise across domains including urban planning, transportation, and epidemic forecasting. Multiplex networks offer another promising structural framework for modelling cross-layer interactions, such as how awareness spreads in a social network and alters behaviour in a mobility and contact network. This enables simulation of co-evolutionary dynamics between information dissemination and infectious disease spreading. Moreover, multiplex networks can explicitly accommodate heterogeneous individual awareness, peer influence, and context-dependent behaviour through both within-network interactions and cross-network dynamics, enabling contagion effects in the information communication layer to be cascaded to influence disease dynamics.
>
> Recent advances in large language models also offer promising capabilities for developing multimodal, self-aware, autonomous multiplex disease models, in which each layer (e.g., mobility, contact, communication) can be designed analogously to GABM agents. However, such models remain largely simulation-based, constrained by the lack of comprehensive, individual-level data records across all network layers. Although such granular and complete datasets are rarely available in reality, emerging data sources may partially address this challenge.
>
> ***Incorporating emerging data sources and robust inference for integrated models***. Novel data streams offer unprecedented opportunities to understand the joint influences of human mobility and social interactions. …

8. The definitions of social network and contact network are unclear. Infectious diseases are transmitted through contact networks. Social network directly affects both physical contacts and information dissemination. The authors should give important definitions at the beginning. I will suggest that the authors just focus on the use of terms in social and contact networks. I wonder whether treating the mobility as a feature that can affect both networks will make the article easier to read. Figure 3 shows that social network affects only information diffusion, and the contact network is more related to movement. This is fine, but the definition should be mentioned earlier and be consistent.

Response [8]:

Thanks for your comments and we understand your confusion. I think we have already defined the two networks in the first two paragraphs of the paper, but their short name may confuse you. We used *contact network* to refer to *human mobility and contact network* for short, which might be a mistake because "mobility" is very important, but we discarded it!

Your understanding is totally correct, that social network consists of social connections facilitating information dissemination and behaviour contagion, while mobility and contact network is more related to human movement. We now describe it clearly in the Introduction, and we used the terms "*social network*" and "*mobility and contact network*" consistently throughout the manuscript.

> … Importantly, human mobility and contact networks (*mobility and contact networks* hereafter), a particular form of geographical network summarising human movement and physical contact, fundamentally shape patterns of infectious disease transmission[1]. As human hosts travel and interact physically, pathogens can be carried and transmitted…
>
> … social interactions and personal relationships (referred to as *social networks*)[11–13]. Within social networks, ties between people, whether through in-person or online interactions, enable knowledge, awareness, and cognition to spread across individual, local and global scales…

However, we are sorry that we cannot use the term social and contact networks you suggested. Detailing the separate roles of social network, mobility and contact network in shaping disease dynamics is one of the key contributions of this review. We should definitely not mix them up. You can find that Chapter 3 "Health behaviour contagion and information dissemination" is entirely about social networks and has nothing to do with mobility and contact, which reason why we need two concepts instead of a unified one.

9. On line 469, what are Big Five personality traits?

Response [9]:

The Big Five personality traits is an important concept in personality psychology. They describe individual differences in five aspects of personality: openness, conscientiousness, extraversion, agreeableness, and neuroticism. These inherent personality traits are closely associated with people's psychological status and behaviours. For more information about it, please refer to:

> McCrae, R. R. & John, O. P. An introduction to the five-factor model and its applications. *J. Pers.* 60, 175–215 (1992).

We mentioned it because it is an indicator used by a cited reference. However, your question made us concerned that it is not a common concept that is easily understood by general readers. Therefore, we decided to remove it in our revised manuscript.

10. On line 484. "In this review, we have highlighted the shared contagion principles governing these networks and identified key research directions to bridge existing knowledge gaps (Table 1)." I am still quite confused about what shared contagion principles have been highlighted. Table 1 said about social media and multiplex networks, but the study did not clearly indicate how many of the included studies address these network types. There should be more studies that cover this topic, see PMID: 38815040 and PMID: 40190382.

Response [10]:

Thanks for your comments and we apologize for the confusion. In the revised manuscript, we have removed the term "shared contagion principles" and describe it more clearly as:

> … In this review, we have highlighted the separate roles of these networks on infectious disease dynamics, summarised existing modelling strategies, and identified key research directions to bridge existing knowledge gaps.

It is followed by our thoughts and suggestions on modelling strategies for future research, which you have already seen in Response [7].

For your second point that how many of the included studies address different network types is unclear, we want to express again our concern that enumeration is not our priority. This scoping review (instead of a systematic review) covers multiple research areas. We can only include several classic/typical/emerging literatures from each area to form our storyline, but cannot list and count all relevant papers. For example, the two paper you mentioned PMID: 38815040 and PMID: 40190382 are definitely valuable reference discussing the impact of misinformation on epidemics. However, misinformation is just a section in our review, and we cannot cite all relevant papers.

Nonetheless, we thank you for pointing out these two important papers and we already cited them in the revised manuscript.

For the Table, you may already see our revision in our previous response, that we have reframed it and included representative reference for each network type.

Again, we thank the reviewers for their suggestions and great help with our manuscript.
